# Factors associated with anxiety and fear of falling in older adults: A rapid systematic review of reviews

**DOI:** 10.1371/journal.pone.0315185

**Published:** 2024-12-18

**Authors:** Carly Whitmore, Sarah Neil-Sztramko, Sebastien Grenier, Amy Gough, Zahra Goodarzi, Erica Weir, Iulia Niculescu, Abitha Suthakaran, Isaac Adedeji, Mahnoor Akram, Juliette Mojgani, Titus Chan, Alastair J. Flint, Heli Juola, Kristin Reynolds, Shanna Trenaman, Michael Van Amerigen, Anthony Yeung, AnneMarie Levy, Andrea Iaboni

**Affiliations:** 1 McMaster University, School of Nursing, Faculty of Health Sciences, Hamilton, Canada; 2 McMaster University, Health Research Methods, Evidence & Impact, Hamilton, Canada; 3 Université de Montréal, Psychology, Montréal, Canada; 4 Dalhousie University, Psychiatry, Halifax, Canada; 5 University of Calgary, Cumming School of Medicine—Geriatrics, Calgary, Canada; 6 Queens University, Medicine and Public Health, Kingston, Canada; 7 Canadian Coalition for Seniors’ Mental Health, Anxiety Guidelines, Toronto, Canada; 8 Department of Psychiatry, Temerty Faculty of Medicine, University of Toronto, Toronto, Canada; 9 University Health Network, Toronto, Canada; 10 Sunnybrook Health Sciences Centre, Psychogeriatric Resources, Toronto, Canada; 11 Department of Psychology, University of Manitoba, Winnipeg, Canada; 12 College of Pharmacy, Faculty of Health, Dalhousie University, Halifax, Canada; 13 Department of Psychiatry and Behavioural Neurosciences, Faculty of Health Sciences, McMaster University, Hamilton, Canada; 14 University of British Columbia, Psychiatry, Vancouver, Canada; University of Maribor, SLOVENIA

## Abstract

**Background:**

Anxiety disorders are prevalent amongst older adults and negatively impact their quality-of-life and health. Anxiety disorders often go undetected or are misattributed to age-related changes. The aim of this systematic review of reviews, was to synthesize existing evidence on risk factors associated with anxiety in older adults to improve opportunities for early detection and intervention.

**Methods:**

A rapid systematic review of reviews was performed. Studies were included if they were systematic reviews, specific to older adults, reported modifiable or non-modifiable factors associated with increased or decreased frequency of anxiety, and reported on anxiety disorders or symptoms of anxiety (including fear of falling).

**Results:**

27 papers met criteria for inclusion. A total of 77 unique risk and protective factors across demographic, health, environmental, and psychosocial domains were identified. Recurrently identified risk factors for anxiety included female sex, health (e.g., multimorbidity, sensory impairments), physical functions (e.g., impaired balance, history of falls), psychological factors (e.g., fear of falling, depression), social isolation, and sleep disturbances, whereas good physical health and balance confidence were protective.

**Conclusions:**

This review reinforces the multifaceted and complex nature of anxiety in older adults. The results synthesized, highlight risk factors that should prompt detection of older adults for anxiety disorders and provide valuable insight for the development of tailored detection tools that better identify older adults at risk. Future research should address methodological limitations and include more diverse populations to improve opportunities for early detection and intervention in this vulnerable population.

## Introduction

Anxiety disorders are the most common mental health problem across all age groups, including in later life. In both community and clinical settings, the prevalence of anxiety disorders amongst older adults varies, and is estimated to range from 1.2% to 17% and 1% to 28%, respectively [1]. Symptoms of anxiety and anxiety disorders, such as generalized anxiety disorder, may have onset in early life and persist throughout the life course, or can be of late onset [2, 3]. Anxiety disorders are more prevalent than depression in later life and can significantly disrupt an older adult’s health, leading to functional impairments, diminished social engagement, increased risk for cognitive impairment, and decreased overall well-being [4, 5].

There are notable barriers to detecting clinically important anxiety in older adults [5]. Despite its prevalence, anxiety in older adults is often overlooked or misattributed to aging (e.g., sleep disturbances, change in cognitive abilities), or conflated with other medical conditions and medication use [6]. Older adults who experience anxiety may exhibit diverse symptoms, ranging from generalized worry, apprehension, and restlessness to lesser recognized age-related concerns focused on health, disability and specific fears, like fear of falling [1, 6, 7]. Fear of falling is particularly pertinent as it can restrict mobility, reduce physical activity, and consequently heighten the risk of falls and related injuries [8]. While older adults (≥ 65) are more likely to correctly recognize the physical symptoms of anxiety, including restlessness and heart palpatations, they are less likely to correctly identify psychological symptoms comapred to younger adults [6, 9]. Furthermore, older adults may downplay psychological symptoms of anxiety and articulate them in different ways than their younger counterparts. Consequently, many older adults living with anxiety go undiagnosed or untreated [10]. This is important, because early detection and treatment of anxiety, either of new anxiety or with the purpose to reduce the intensity of existing anxiety, has been found to increase quality of life and prevent unfavourable outcomes [10].

One way to improve the detection of anxiety in older adults is to recognize those who may be at risk. This includes a need to identify those demographic, health, and psychosocial factors that can increase risk, or may be protective of anxiety and anxiety symptoms in this population. Previous reviews on these factors have provided valuable insights into the complex interplay between various determinants and the development or exacerbation of anxiety in older adults. However, many recent reviews have focused on specific anxiety disorders or have limited their scope to distinct subpopulations of older adults (e.g., those with specific chronic illness or cognitive decline). Given the breadth of literature, an overarching synthesis is needed to identify commonalities across these individual reviews and provide a more comprehensive understanding of the risk and protective factors for anxiety in older adults. Considering the large body of literature available, there is a need for a synthesis of this literature to identify commonalities in these factors for anxiety across older adult populations.

This rapid review of reviews responds to this gap by synthesizing findings from multiple systematic reviews to offer a high-level overview of patterns across diverse studies. This approach is valuable because it consolidates fragmented evidence, highlights areas of agreement and inconsistency, and addresses the limitations of traditional systematic reviews that often focus narrowly on specific disorders or populations. This review aims to support clinicians in more effective case finding and intervention for anxiety in older adults.

The aim of this review of reviews was to synthesize the available evidence on risk factors for anxiety in older adults. This rapid review was completed as part of the development of guidelines for the assessment and treatment of anxiety [11]. A rapid review is a sub-type of the systematic review in which some components are either simplified or omitted with the purpose of producing a synthesis of available evidence [12, 13]. This review was guided by the question, “What are the factors associated with anxiety in older adults?”

## Methods

Our rapid review approach followed the recommendations outlined by Tricco et al. [13]. No protocol for this rapid review was registered. Streamlined methods used in this review included: limiting the literature search to peer-reviewed articles in 3 databases (PsycInfo, Embase, and Medline), no hand searching, limiting inclusion criteria to English and French language, and presenting results in a narrative summary. The Canadian Coalition for Seniors’ Mental Health Anxiety Guidelines working group guided the development of the research question, search terms, and extraction plan. This manuscript follows the Preferred Reporting Items for Systematic Reviews and Meta-Analyses Checklist (see S1 Table. PRISMA checklist).

### Study selection

The database search was completed in February 2023 and updated in April 2024 (see S1 File. Search strategy and terms). The search strategy was developed in consultation with a health sciences librarian and informed by the clinical expertise of the authorship team. Published articles were included if: they were systematic reviews, specific to an older adult population, including if 80% of the studies include a sample aged ≥65 or results specific to older adults are presented separately, reported modifiable or non-modifiable factors associated with increased or decreased frequency of anxiety, and reported on anxiety disorders or symptoms of anxiety including fear or fear of falling. Studies were excluded from this review if: they were interventional or qualitative in nature, not specific to an older adult population, or reported on factors associated with mental health conditions that are no longer categorized as anxiety disorders in the Diagnostic and Statistical Manual of Mental Disorders, fifth edition (DSM-5; e.g., post-traumatic stress disorder, obsessive compulsive disorder).

**Fig 1:**
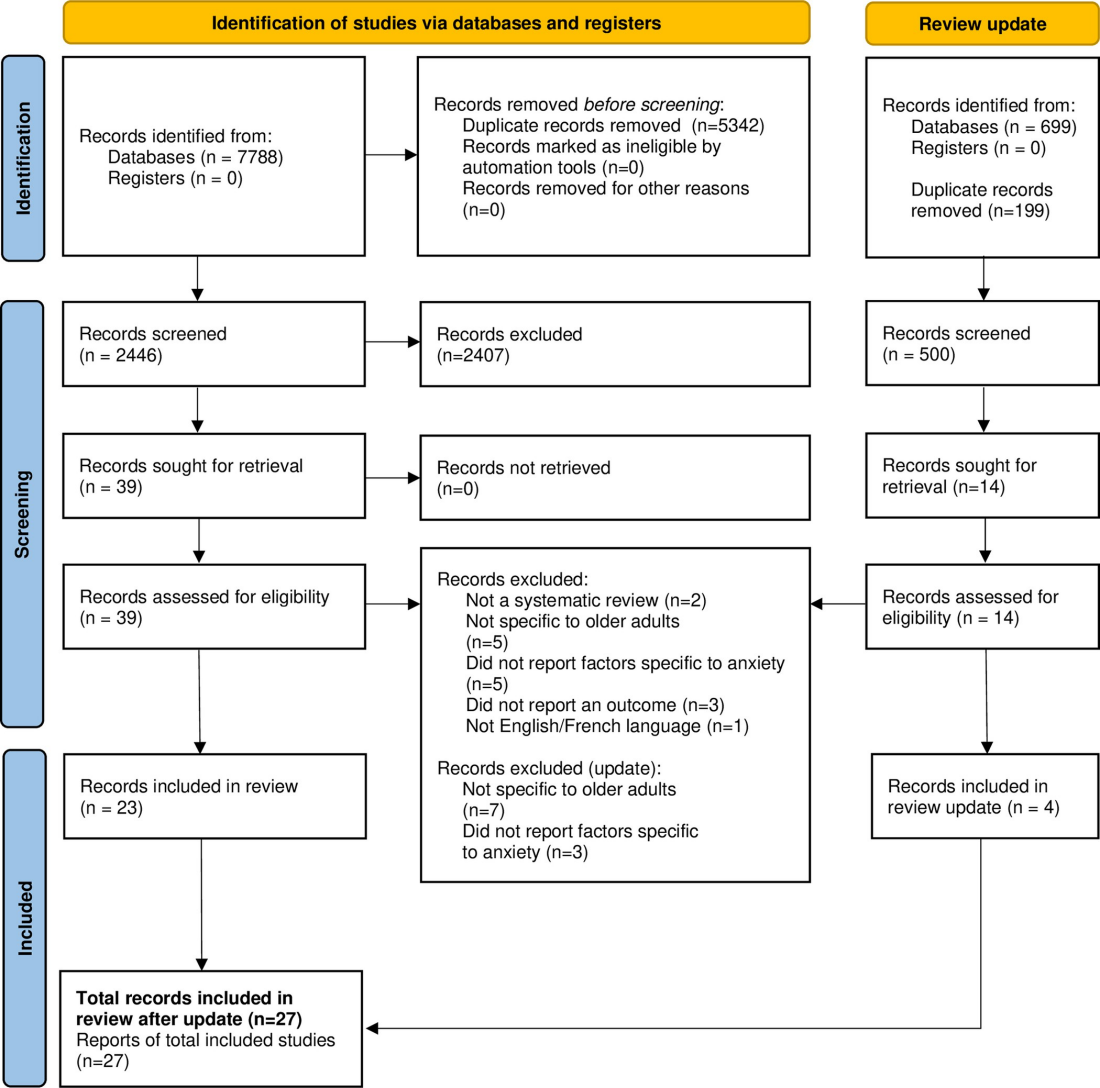
PRISMA diagram.

Screening and exclusion was completed by two independent reviewers using Distiller SR. Full-text articles were similarly reviewed by two independent reviewers. Disagreements were resolved through consensus and reasons for exclusion were recorded. A summary of study selection and PRISMA diagram is provided (Fig 1).

### Data abstraction

Data from each of the included articles were abstracted to a template by two independent reviewers. This template included the aim of the review, the type and number of studies included, how older adults were defined, the mean age of participants, the number of female participants, how anxiety was measured or assessed, and any factors described to be risks or preventative of anxiety. In this review, we did not report on data points that were missing from the included studies and focused solely on available information.

#### Quality appraisal

All included reviews were subject to a quality appraisal using the Measurement Tool to Assess systematic Reviews (AMSTAR 2) tool (see S2 Table. Quality appraisal of the included reviews using the AMSTAR 2) [14, 15]. Quality assessments were completed by two independent reviewers and any disagreements were resolved through consensus. No articles were eliminated during the quality appraisal phase.

### Data synthesis

Abstracted data (see S3 Table. Full export of extracted data; see S4 Table. Excluded studies and reasons; see S5 Table. All studies identified in search) were analyzed using a data-driven, content analytic approach aligned with the descriptive nature of the review. After reading and re-reading the abstracted data, data were summarized and presented to the Canadian Coalition for Seniors’ Mental Health anxiety working group for discussion, interpretation, and consensus.

## Results

### Characteristics of included studies

Of the 27 reviews included in this rapid review, 10 included a meta-analysis (see Table 1). Many included reviews described the population of interest using an age-based definition (n = 14), including adults aged 60 and older (n = 12), or those that simply described their sample as “older adults” (n = 2). Further definitions of the sample included a focus on a specific disease or health condition, such as cognitive impairment or dementia (n = 2), or cancer (n = 4), while others had a geographical or location-based definition like residential care (n = 1) or a community-dwelling sample (n = 2). Most reviews focused on identifying factors associated with anxiety disorders or symptoms of anxiety (n = 19), while some focused on death anxiety (n = 2) or fear of falling (n = 8). However, these foci were not mutually exclusive.

**Table 1:** Summary characteristics of the 27 reviews included in the rapid review.

Author, Year	Aim of review (meta-analysis noted)	Sample population	Anxiety focus	Number (n) of studies included	Anxiety tools	Last search date
Cheng, 2019 [16]	To synthesize the existing literature on the prevalence and predictors of anxiety and depression in near-centenarians and centenarians	Adults 95 years and older	Anxiety	Cross-sectional (n = 6)	Brief Symptom Inventory DSM diagnosis HADS LEIPAD	July 2018
Cipriani, 2021 [17]	To examine the relationship between sleep problems and psychological distress during the COVID-19 pandemic	Adults aged 60 years and older	Anxiety	Cohort (n = 11)	Kessler Psychological Distress scale	July 2021
Ciuffreda, 2021 [18]	To explore the factors associated with depression and anxiety during the COVID-19 pandemic	Adults aged 60 years and older	Anxiety	Cohort (n = 1) Cross-sectional (n = 10)	General Anxiety Disorder scale (GAD-7) HADS Depression, Anxiety, and Stress Scale (DASS-21)	February 2021
Coelho-Júnior, 2022 [19]	To explore the association between religious and spiritual practices and mental health in older adults Meta-analysis	Adults aged 60 years and older	Anxiety Death anxiety	Not reported (n = 102)	Beck Anxiety Inventory Death Anxiety Scale	July 2021
Creighton, 2017 [20]	To synthesize and summarize studies examining the correlates and predictors of anxiety in older adults living in residential aged care	Older adults living in residential aged care	Anxiety	Not reported (n = 34)	HADS Social Avoidance and Distress Scale Geriatric Anxiety Scale Rating Anxiety in Dementia scale Hamilton Anxiety Rating Scale (HARS)	November 2015
Denkinger, 2015 [21]	To identify and analyse predictors of fear of falling (update to Scheffer et al., 2008)	Community-dwelling older adults	Fear of falling	Not reported (n = 20)	Consequence of Falling scale Fear of falling activity restriction scale Falls Efficacy Scale (FES)	October 2013
Fonseca, 2021 [22]	To assess the correlation between pain severity and depressive and anxious symptomatology in older adults with osteoarthritis Meta-analysis	Older adults living with osteoarthritis	Anxiety	Cohort (n = 7) Case control (n = 1) Cross-sectional (n = 113)	HADS State-Trait Anxiety Inventory GAD-7 Patient Reported Outcomes Measurement Information System DASS-21 Beck Anxiety Inventory	January 2020
Gambaro, 2022 [23]	To describe the association between depressive symptomatology and antidepressant therapy on fear of falling Meta-analysis	Adults aged 60 years and older	Fear of falling	Cohort (n = 12) Cross-sectional (n = 5) Observational (n = 1)	Modified FES	July 2020
Grenier, 2019 [24]	To provide an estimate of prevalence of anxiety disorders in older adults and to identify the impact of gender and age Meta-analysis	Older adults	Anxiety	Not reported (n = 38)	GAD-7 Anxiety and Related Disorders Interview Schedule	December 2016 (updated April 2018)
Guerra, 2024 [25]	To investigate factors contributing to concerns about falling and activity restriction in the community among older adults who had a hip fracture	Adults aged 50 years and older	Fear of falling	Cohort (n = 9) Qualitative (n = 9) Mixed methods (n = 1)	Activities-specific Balance Confidence scale FES FES-International	June 2022
Han, 2024 [26]	To evaluate the current literature on frailty, symptoms, and health-related quality of life, and examine the associations of frailty with these factors in older adult cancer survivors Meta-analysis	Old adult cancer survivors aged 60 years and older	Anxiety	Cohort (n = 16) Cross-sectional (n = 10)	HADS GAD Geriatric Depression Scale Clinical Symptom Inventory	February 2023
Hwang, 2020 [27]	To examine factors related to anxiety in persons with dementia and identify potentially modifiable factors in the same population	Adults living with dementia	Anxiety	Cohort (n = 6) Cross-sectional (n = 18)	Rating Anxiety in Dementia scale HADS HARS Philadelphia Geriatric Center Apparent Affect Rating Scale Behavioral Pathology in Alzheimer’s Disease Rating Scale	July 2019
Kang, 2022 [28]	To explore the relationship between ageism and psychological well-being of older adults	Adults aged 60 years and older	Anxiety	Cohort (n = 2) Cross-sectional (n = 11)	DASS-21	August 2019
Lee, 2023 [29]	To evaluate the risk factors of depression and anxiety in older adults with cancer Meta-analysis	Adults with cancer	Anxiety	Cohort (n = 33)	HADS International Classification of Diseases diagnosis Psychosocial Screen for Cancer	Not reported
Pai, 2019 [30]	To review the illness perceptions of stroke patients and to examine the association between illness representation and psychological distress Meta-analysis	Adults who have had a stroke	Anxiety	Cross-sectional (n = 4) Correlational (n = 1) Longitudinal (n = 2)	HADS Recovery Locus of Control scale Ways of Coping scale	October 2018
Parpa, 2015 [31]	To examine the relationship between aging, cancer, and psychiatric disorders or psychological problems in older adults with cancer	Adults with cancer who are 65 years and older	Anxiety Death anxiety	Not reported (n = 102)	HADS HARS	Not reported
Payette, 2016 [32]	To summarize the relationship between anxiety and fear of falling and other fall-related concerns among community-dwelling older adults Meta-analysis	Community-dwelling older adults aged 65 years and older	Fear of falling	Cohort (n = 2) Case control (n = 3) Cross-sectional (n = 14) Pre/post-test (n = 1)	Geriatric Anxiety Inventory HARS GAD-7 Geriatric Anxiety Scale HADS	June 2015
Sagna, 2014 [33]	To examine common and distinguishing factors associated with the co-occurrence of depression and anxiety disorders among older adults with Parkinson’s Disease.	Adults with Parkinson’s disease who are 60 years and older	Anxiety	Case control (n = 4) Cross-sectional (n = 1)	State-Trait Anxiety Inventory (STAI) HARS	March 2013
Scheffer, 2008 [34]	To identify factors related to fear of falling in community-dwelling older adults	Community-dwelling older adults	Fear of falling	Cross-sectional (n = 20) Prospective (n = 8)	Self-report Activity related measures	Not reported
Silva, 2022 [35]	To summarize literature on the relationship between sociodemographic, clinical, and psychosocial factors and emotional distress in older adults with cancer	Older adults with cancer	Anxiety	Cohort (n = 5) Cross-sectional (n = 15)	GAD-7	December 2021
Tan, 2023 [36]	To investigate the association between frailty and anxiety	Older adults aged 60 years and older	Anxiety	Cohort (n = 1) Cross-sectional (n = 20)	HADS GAD STAI PROMIS Geriatric Anxiety Inventory DSM-5	October 2021
Vink, 2008 [37]	To examine risk factors of anxiety, anxiety symptoms, and depression in older adults	Older adults aged 50 years and older	Anxiety	Cohort (n = 5) Cross-sectional (n = 12)	DSM-5 or International Classification of Diseases diagnosis Self-report symptoms	Not reported
Visla, 2022 [38]	To examine the relationship between worry and various mental health indicators Meta-analysis	Older adults	Anxiety	Not reported (n = 120)	GAD-7 STAI HARS HADS	Not reported
Visschedijk, 2010 [39]	To describe and analyze factors associated with fear of falling in individuals after hip fracture	Adults who have had a hip fracture	Fear of falling	Cohort (n = 6) Cross-sectional (n = 3) Randomized trial (n = 4) Descriptive (n = 1)	Activities-specific Balance Confidence scale FES Self-report	March 2009
Vo, 2023 [40]	To describe factors related to fear of falling among older adults in Southeast Asia	Older adults living in Southeast Asia	Fear of falling	Cohort (n = 1) Case control (n = 1) Cross-sectional (n = 13) Experimental (n = 3)	FES-I Self-report	Not reported
Xiong, 2023 [41]	To explore risk factors of fear of falling among older adults Meta-analysis	Older adults aged 60 years and older	Fear of falling	Not reported (n = 153)	FES-I SFES-I Modified Fall Efficacy Scale	September 2023
Yates, 2013 [42]	To explore the relationship between mild cognitive impairment and mood or anxiety disorder	Adults with mild cognitive impairment	Anxiety	Cohort (n = 3) Cross-sectional (n = 25) Longitudinal (n = 30)	Goldberg Anxiety Disorder Scale STAI Geriatric Anxiety Inventory Anxiety Sensitivity Index	Not reported

### Anxiety assessment

Across these reviews, 32 different anxiety tools and scales were used in the included studies. Commonly used tools and scales included the Hospital Anxiety and Depression Scale (n = 9), the General Anxiety Disorder scale (n = 8), the Hamilton Anxiety Rating Scale (n = 6), and the Depression, Anxiety, and Stress Scale (n = 3).

### Risk and protective factors

In the 27 included papers, 77 unique risk and protective factors for anxiety and fear of falling were identified (see Table 2). These factors spanned diverse categories, including demographic variables, objective health measures, physical function outcomes, medication use, social and environmental factors, sleep outcomes, psychological, and neurological factors. While some of the 77 factors were only reported once in a single review, some commonly reported risk factors included: female sex (n = 9); functional limitations (n = 8); multimorbidity (n = 7); history of falls (n = 5); impaired balance (n = 5); pain (n = 5); cognitive impairment (n = 4); low self-rated health (n = 4); loneliness or social isolation (n = 5); low social support (n = 4); depression (n = 3); and sleep disturbance or insomnia (n = 3).

**Table 2:** Synthesis of risk factors, protective factors, and factors reported to have no association with anxiety.

Factor	No Association	Protective	Risk	Studies
**Demographic**				
Age	x			Cheng, 2019 Ciuffreda, 2021 Creighton, 2017 Silva, 2022
Younger age			x	Parpa, 2015
Older age			x	Scheffer, 2008^α^ Vo, 2023^α^ **Xiong, 2024**^α^
		x		**Lee, 2023** Parpa, 2015
Education	x			Ciuffreda, 2021 Creighton, 2017
Higher Education	x			**Lee, 2023**
		x		**Lee, 2023**
Lower Education			x	**Xiong, 2024** ^α^
Sex	x			Cheng, 2019
Female Sex			x	Ciuffreda, 2021 Creighton, 2017 Denkinger, 2015^α^ **Grenier, 2019** **Lee, 2023** Scheffer, 2008^α^ Vink, 2008 Vo, 2023^α^ **Xiong, 2024**^α^
**Health Related**				
Alcohol consumption	x			Ciuffreda, 2021
Cancer treatment	x			Silva, 2022
Cancer type	x			Silva, 2022
Good physical health		x		Hwang, 2020
High blood pressure			x	Vink, 2008
High body mass index			x	**Xiong, 2024** ^α^
History of falls			x	**Payette, 2016**^α^ Scheffer, 2008^α^ Visschedijk, 2010^α^ Vo, 2023^α^ **Xiong, 2024**^α^
High self-rated health		x		Visla, 2022
Low self-rated health			x	Cheng, 2019 Scheffer, 2008^α^ Vink, 2008 **Xiong, 2024**^α^
Multimorbidity	x			Vink, 2008 **Xiong, 2024**^α^
			x	Creighton, 2017 Guerra, 2024^α^ **Han, 2024** **Lee, 2023** Silva, 2022 Vink, 2008 Vo, 2023^α^
Pain			x	Creighton, 2017 Fonseca, 2022 Parpa, 2015 Silva, 2022 Xiong, 2024^α^
Poor health status	x			Vink, 2008
Poor objective health status			x	Cheng, 2019
Smoking status	x			Ciuffreda, 2021
Vision and hearing loss or impairment			x	Vink, 2008 **Xiong, 2024**^α^
**Physical Function**				
Activity restrictions			x	Visschedijk, 2010^α^ **Xiong, 2024**^α^
Frailty			x	Tan, 2023 **Xiong, 2024**^α^
Functional limitations	x			Cheng, 2019 Vink, 2008
			x	Creighton, 2017 Denkinger, 2015^α^ Guerra, 2024^α^ **Han, 2024** Scheffer, 2008^α^ Vink, 2008 Visschedijk, 2010^α^ Vo, 2023^α^
Gait challenges	x			**Gambaro, 2022**
			x	Visschedijk, 2010^α^ Vo, 2023^α^
Impaired balance			x	Denkinger, 2015^α^ **Gambaro, 2022** Scheffer, 2008^α^ Vo, 2023^α^ **Xiong, 2024**^α^
Use of walking aid			x	Denkinger, 2015^α^ **Xiong, 2024**^α^
**Medication Use**				
Androgen deprivation therapy			x	**Lee, 2023**
Anticholinergics, antipsychotics, or antidepressants			x	Creighton, 2017
Functional dependence	x			Creighton, 2017
Polypharmacy	x			Denkinger, 2015^α^
			x	Creighton, 2017
**Social and Environmental**				
Accessibility (geography)			x	Guerra, 2024^α^
Access to green space	x			Ciuffreda, 2021
Caregiver stress			x	Hwang, 2020
Experience of ageism			x	Kang, 2022
Experience of discrimination			x	Kang, 2022
High spirituality		x		**Coelho-Junior, 2022**
Intrinsic religiosity		x		**Coelho-Junior, 2022**
Lack of access to environmental adaptations			x	Guerra, 2024^α^
Lower caregiver competence			x	Hwang, 2020
Low income			x	Cheng, 2019 Scheffer, 2008^α^
Low social support			x	Creighton, 2017 Guerra, 2024^α^ **Lee, 2023** Vo, 2023^α^
	x			**Xiong, 2024** ^α^
Loneliness			x	Cheng, 2019 Cipriani, 2021 Ciuffreda, 2021
Living alone			x	**Lee, 2023** **Xiong, 2024** ^α^
Religious affiliation		x		**Coelho-Junior, 2022**
Rural living	x			Ciuffreda, 2021
Social isolation			x	**Han 2024** Hwang, 2020
Unmarried, divorced, separated			x	**Lee, 2023**
	x			**Xiong, 2024** ^α^
Unmet care needs			x	Creighton, 2017
**Sleep**				
Insomnia			x	Ciuffreda, 2021
Sleep disturbance			x	Cipriani, 2021 Creighton, 2017
**Psychological**				
Balance confidence		x		**Payette, 2016** ^α^
Better quality of life		x		Hwang, 2020
Depression			x	**Gambaro, 2022** Scheffer, 2008^α^ Vo, 2023^α^
Fear of falling			x	Creighton, 2017 **Payette, 2016**^α^
High level of stress			x	Cipriani, 2021
Hope	x			Creighton, 2017
Life satisfaction		x		**Coelho-Junior, 2022**
Lower quality of life			x	Creighton, 2017
Meaning in life		x		**Coelho-Junior, 2022**
Other psychiatric illness	x			Ciuffreda, 2021
Positive affect		x		Guerra, 2024^α^
Positive attachment		x		Hwang, 2020
Personal belief condition (stroke) can be controlled	x			Pai, 2019
Personal belief there is a cure to condition (stroke)		x		Pai, 2019
Poor perception about rehabilitation at hospital or home			x	Guerra, 2024^α^
Worry about falls	x			Visschedijk, 2010^α^
			x	Visschedijk, 2010^α^
**Neurological**				
Akinetic or tremor symptoms	x			Sagna, 2014
Autonomic symptoms			x	Sagna, 2014
Cognitive Impairment	x			Vink, 2008
			x	Scheffer, 2008^α^ Vink, 2008 Vo, 2023^α^ Yates, 2013
Dementia diagnosis			x	Hwang, 2020
Motor fluctuations			x	Sagna, 2014
Parkinson’s disease severity	x			Sagna, 2014
Parkinson’s disease duration	x			Sagna, 2014
			x	Sagna, 2014
Reduction in gray matter			x	Hwang, 2020
Severity of motor symptoms			x	Sagna, 2014
Subjective cognitive decline			x	Ciuffreda, 2021
Vascular dementia			x	Hwang, 2020
Younger age of Parkinson’s disease onset	x			Sagna, 2014
			x	Sagna, 2014

^α^ Focus of review is on the fear of falling

Bold = meta-analysis

In addition to these broad factors, there were a number of discrepant findings reported in the included reviews, with some of these factors overlapping with those which were commonly reported. This included age, cognitive impairment, the presence of functional limitations, including gait challenges, level of education, polypharmacy, worry about falls, and the presence of multiple chronic conditions. For example, in the reviews by Cheng et al. (2019), Ciuffreda et al., (2021), Creighton et al. (2017), and Silva (2022), age had no association with anxiety. Two studies of older adults with cancer found that older age was associated with less risk of anxiety (Lee et al., (2023); Parpa et al., (2015). Whereas in the reviews by Scheffer et al., (2008), Vo et al., (2023), and Xiong et al. (2024), older age was a risk factor for fear of falling, specifically.

Only one factor, older age, was described to be protective of anxiety in this population and was reported more than one time across the included reviews. Of the protective factors identified, many can be considered psychological factors, including life satisfaction, positive affect, positive attachment, better quality of life, and reporting meaning in life. There were also a number of spiritual or religious factors, including high spirituality, intrinsic religiosity, and religious affiliation that were reported to be protective of anxiety in this population.

## Discussion

In this rapid review of reviews, we have comprehensively summarized a wide array of factors associated with anxiety and fear of falling in older adults, culminating in the identification of 77 unique risk and protective factors. These findings, particularly the number of factors as well as the discrepancies noted, underscore the multifaceted nature of anxiety in this demographic, shaped by demographic, health, environmental, and psychosocial influences. Contrary to common misconception, anxiety in older adults is not solely a consequence of aging, rather, it is influenced by a complex interplay of factors that demand nuanced consideration [43].

Anxiety and fear of falling in older adults may be present as a result of a combination of factors rather than one single factor. Female sex, functional limitations, multimorbidity, history of falls, impaired balance, pain, cognitive impairment, low self-rated health, loneliness or social isolation, low social support, depression, and sleep disturbance or insomnia were among those risk factors recurrently identified. These factors may heighten feelings of vulnerability and risk (e.g., loneliness, pain), amplify concerns about independence and ability (e.g., functional limitations, gait challenges), or even further intensify feelings of existing anxiety or fear of falling (e.g., sleep disturbance). These findings align with previous research and suggest that interventions for anxiety in older adults may need to target multiple domains of health and wellbeing.

Importantly, many of the factors identified can be considered modifiable. Considering that most older adults living with anxiety and anxiety symptoms have been doing so for most of their lives, understanding the ways that these multifaceted factors present is critical in designing comprehensive interventions and support systems that address the needs of this population. Integrative approaches focusing on social support, healthcare accessibility, behavioral modifications, and mental health management can significantly improve the well-being of these older adults.

Screening for anxiety is most effective when it is targeted at those older adults who are at a higher risk [3, 6, 11]. The presence of the factors described in this paper may prompt further consideration of a screening tool to support timely case finding or to further inquire about symptoms that meet diagnostic criteria. Recognizing and acting upon these factors through more fulsome assessment and testing can lead to tailored interventions that better support and enhance the mental health of older adults affected by anxiety [44]. Moreover, there’s a clear need for further research, employing standardized methodologies and assessments, to deepen our understanding of the intricate relationship between these multifarious factors and anxiety in older adults, enabling more targeted and effective interventions.

When considering the high prevalence of anxiety in older adults, acknowledging the multifaceted nature of anxiety and fear of falling in older adults presents an opportunity to promote equity, implement tailored and personalized biopsychosocial intervention, and foster a more holistic approach to well-being [6, 8]. One way to do this is to consider these broad and multifarious factors in the context of the social and structural determinants of health and the ways that these factors span various domains of life [45–47]. This includes socioeconomic factors (e.g., low income, level of education); psychosocial factors (e.g., marital status or living arrangement, social support, caregiving status), biological and genetic factors (e.g., age, sex, presence of chronic disease), and identity and experiential factors (e.g., experience of discrimination, racism, or sexism). While the biological determinants of health may continue to dominate in certain spaces, there is an opportunity for health and social care providers and professionals to act upon these social and structural determinants of health. A comprehensive understanding of the impact of these factors on health can ultimately result in more effective treatment, improved screening, and timely referrals [47]. There is a need for further research to explore upstream public health interventions that aim to address social and structural determinants of health, enhance protective factors, and prevent anxiety in older adults.

### Limitations

Findings presented in this rapid review are limited by the evidence synthesized, the review design, and the underrepresentation of certain populations in the evidence identified. First, while this synthesis describes factors identified as protective or of risk for anxiety in older adults, directionality or causality cannot be discerned. For example, while cognitive impairment, depression, and insomnia are described as risk factors, we also have evidence that there is a bidirectional relationship between these factors and anxiety. It is also important to acknowledge that this review is confined to synthesized evidence from existing reviews, constituting a limitation in scope. The exclusion of primary studies and the nature of a rapid review may have restricted the depth of insights gleaned from individual studies, necessitating caution in interpreting the findings comprehensively. No de-duplication of studies was completed, although based on the number of factors that were reported by only one study, the unique focus of included reviews on sub-groups of older adults, including those of specific age, conditions, or locations, and that vote-counting is not used to indicate strength of association, this risk is limited. Further, because many papers did not discern between anxiety disorder and subsyndromal symptoms of anxiety, it is unknown whether there are differences in associated factors.

Secondly, it was found that the quality of papers included in this review were quite low. Critical domain flaws were identified for each paper, and for many papers, were significant. These limitations underscore the necessity for enhanced methodological rigour in review reporting. Strengthening the quality of this evidence would bolster the reliability and robustness of conclusions drawn in this review of reviews.

Lastly, this review is limited by underrepresentation of specific populations in included studies. The absence of studies focused on indigenous populations, racialized or gender diverse groups or those who experience incarceration or institutionalization restricts the generalizability and applicability of the findings to these groups. Further, there is a limited focus on the varied experience of older adults during older adulthood. Considering that this period can span upwards of four decades, there is a need to better understand the differences and commonalities in factors present in the oldest old compared to youngest old, for example. Additionally, it is crucial to acknowledge that some groups, such as those who experience discrimination or racism, are at heightened risk of negative mental health outcomes related to their experiences, underscoring the importance of considering intersectional factors in research and interventions targeting anxiety in older adults. There is a need for future research to encompass a more expansive and inclusive approach to understand anxiety and factors that are associated with anxiety across varied groups within older adult populations.

## Conclusion

This rapid review provided a foundational step in the development of key clinical practice guidelines to treat and manage anxiety in older adults. Through identifying factors described as associated with anxiety and fear of falling in this population, this rapid review lays the groundwork for informed and targeted interventions. The comprehensive insights gleaned from the diverse range of risk and protective factors identified, provides valuable guidance for healthcare professionals, policymakers, and researchers. Future research should build on these findings by developing theoretical models that further explore the interplay between these factors and by testing interventions that address both clinical and social determinants of anxiety in older adults.

## Supporting information

S1 Table PRISMA checklist. (DOCX)

S2 Table Quality appraisal of the included reviews using the AMSTAR 2. (DOCX)

S3 Table Full export of extracted data. (XLSX)

S4 Table Excluded studies and reasons. (XLSX)

S5 Table All studies identified in search. (XLSX)

S1 File Search strategy and terms. (PDF)
