# Supplementary material for: Factors associated with anxiety and fear of falling in older adults: A rapid systematic review of reviews
Source: PLoS One. 2024 Dec 18;19(12):e0315185. doi: 10.1371/journal.pone.0315185 (PMC11654959; doi:10.1371/journal.pone.0315185)
Supplement: S2 Table — (DOCX) [file pone.0315185.s002.docx]

| **AMSTAR 2 Items** | **Cheng, 2019** | **Cipriani, 2021** | **Ciuffreda, 2021** | **Coelho-Junior, 2022*** | **Creighton, 2017** | **Denkinger, 2015**^α^ | **Fonseca, 2022*** | **Gambaro, 2022*** | **Grenier, 2019**^α^***** | **Guerra, 2024**^α^ | **Han, 2024*** | **Hwang, 2020** | **Kang, 2022** | **Lee, 2023*** | **Pai, 2019*** | **Parpa, 2015** | **Payette, 2016**^α^***** | **Sagna, 2014** | **Scheffer, 2008**^α^ | **Silva, 2022** | **Tan, 2023** | **Vink, 2008** | **Visla, 2022*** | **Visschedijk, 2010**^α^ | **Vo, 2023**^α^ | **Xiong, 2024**^α^***** | **Yates, 2013** |
| --- | --- | --- | --- | --- | --- | --- | --- | --- | --- | --- | --- | --- | --- | --- | --- | --- | --- | --- | --- | --- | --- | --- | --- | --- | --- | --- | --- |
| Research questions and inclusion criteria for the review include the components of PICO |  |  |  |  |  |  |  |  |  |  |  |  |  |  |  |  |  |  |  |  |  |  |  |  |  |  |  |
| Review methods established prior to the review and deviations from  protocol were justified |  |  |  |  |  |  |  |  |  |  |  |  |  |  |  |  |  |  |  |  |  |  |  |  |  |  |  |
| Authors explain their selection of the study designs for inclusion in the review |  |  |  |  |  |  |  |  |  |  |  |  |  |  |  |  |  |  |  |  |  |  |  |  |  |  |  |
| Authors use a comprehensive literature search strategy |  |  |  |  |  |  |  |  |  |  |  |  |  |  |  |  |  |  |  |  |  |  |  |  |  |  |  |
| Authors perform study selection in duplicate |  |  |  |  |  |  |  |  |  |  |  |  |  |  |  |  |  |  |  |  |  |  |  |  |  |  |  |
| Authors perform data extraction in duplicate |  |  |  |  |  |  |  |  |  |  |  |  |  |  |  |  |  |  |  |  |  |  |  |  |  |  |  |
| Authors provide a list of excluded studies and justify the exclusions |  |  |  |  |  |  |  |  |  |  |  |  |  |  |  |  |  |  |  |  |  |  |  |  |  |  |  |
| Authors describe the included studies in adequate detail |  |  |  |  |  |  |  |  |  |  |  |  |  |  |  |  |  |  |  |  |  |  |  |  |  |  |  |
| Authors use a satisfactory technique for assessing the risk of bias |  |  |  |  |  |  |  |  |  |  |  |  |  |  |  |  |  |  |  |  |  |  |  |  |  |  |  |
| Authors report on the sources of funding for the studies included in the review |  |  |  |  |  |  |  |  |  |  |  |  |  |  |  |  |  |  |  |  |  |  |  |  |  |  |  |
| For RCT, if meta-analysis used, authors use appropriate methods for statistical combination of results |  |  |  |  |  |  |  |  |  |  |  |  |  |  |  |  |  |  |  |  |  |  |  |  |  |  |  |
| For NSRI, if meta-analysis used, authors use appropriate methods for statistical combination of results |  |  |  |  |  |  |  |  |  |  |  |  |  |  |  |  |  |  |  |  |  |  |  |  |  |  |  |
| Authors provide a satisfactory explanation for, and discussion of, any heterogeneity observed |  |  |  |  |  |  |  |  |  |  |  |  |  |  |  |  |  |  |  |  |  |  |  |  |  |  |  |
| If quantitative synthesis used, authors perform investigation of publication bias (small study bias) |  |  |  |  |  |  |  |  |  |  |  |  |  |  |  |  |  |  |  |  |  |  |  |  |  |  |  |
| Authors report conflict of interest, including any funding received for conducting the review |  |  |  |  |  |  |  |  |  |  |  |  |  |  |  |  |  |  |  |  |  |  |  |  |  |  |  |

^α^ *Focus of review is on the fear of falling.*

** Meta-analysis conducted.*

Quality appraisal decisions on the AMSTAR 2 items for included reviews. Cells shaded green denote a “Yes” response, cells shaded magenta
 denote a “No” response, cells shaded yellow denote a “Partial Yes”, and cells shaded gray indicate no meta-analysis was conducted.
 Randomized controlled trial (RCT), Non-randomized study of interventions (NSRI)
