# Supplementary material for: Factors associated with anxiety and fear of falling in older adults: A rapid systematic review of reviews
Source: PLoS One. 2024 Dec 18;19(12):e0315185. doi: 10.1371/journal.pone.0315185 (PMC11654959; doi:10.1371/journal.pone.0315185)
Supplement: S1 File — (PDF) [file pone.0315185.s006.pdf]

Supplemental File 1: Search Strategy and Terms

**MEDLINE**	Search Statement
1.	exp Anxiety/
2.	(anxiety or anxieties or anxious or anxiousness).tw,kf.
3.	Anxiety Disorders/
4.	anxiety disorder?.tw,kf.
5.	anxiety state?.tw,kf.
6.	Panic Disorder/
7.	(panic adj (disorder? or attack?)).tw,kf.
8.	Agoraphobia/
9.	Agoraphobia?.tw,kf.
10.	exp Phobic Disorders/
11.	claustrophobia?.tw,kf.
12.	(phobia? or phobic).tw,kf.
13.	Phobia, Social/
14.	social anxiety disorder?.tw,kf.
15.	(fear* adj3 fall*).tw,kf.
16.	or/1-15
17.	aging/
18.	aged/
19.	"aged, 80 and over"/
20.	"frail elderly"/
21.	"health services for the aged"/
22.	"homes for the aged"/
23.	geriatrics/
24.	((old or retired) adj2 (people* or patient* or inpatient* or in-patient* or outpatient* or out-patient* or client* or person* or individual* or wom#n or man or men or age)).tw,kw.
25.	(aging or ageing or elder* or older* or geriatr* or gerontolog* or senior* or senescen* or retiree* or sexagenarian* or septuagenarian* or octagenarian* or nonagenarian* or centenarian* or supercentenarian* or veteran*).tw,kw,jw.
26.	(aged care or aged adult? or aged people or aged patient? or aged person? or aged women or aged men or aged individual?).tw,kf.

27.	or/17-26
28.	16 and 27
29.	Risk factor.mp. or exp Risk Factors/
30.	exp Risk Factors/
31.	((prevention or risk or protect* or mediat* or moderat*) adj2 (assess* or factor*)).tw,kw.
32.	exp Risk assessment/
33.	risk stratification.mp.
34.	risk modelling.tw.
35.	risk factors.tw.
36.	(predict* or influenc* or determinant* or associat* or correlat*).tw,kw.
37.	or/29-36
38.	(systematic review or meta-analysis).pt.
39.	meta-analysis/ or systematic review/ or systematic reviews as topic/ or meta-analysis as topic/ or "meta analysis (topic)"/ or "systematic review (topic)"/ or exp technology assessment, biomedical/ or network meta-analysis/
40.	((systematic* adj3 (review* or overview*)) or (methodologic* adj3 (review* or overview*))).ti,ab,kf.
41.	((quantitative adj3 (review* or overview* or synthes*)) or (research adj3 (integrati* or overview*))).ti,ab,kf.
42.	((integrative adj3 (review* or overview*)) or (collaborative adj3 (review* or overview*)) or (pool* adj3 analy*)).ti,ab,kf.
43.	(data synthes* or data extraction* or data abstraction*).ti,ab,kf.
44.	(handsearch* or hand search*).ti,ab,kf.
45.	(mantel haenszel or peto or der simonian or dersimonian or fixed effect* or latin square*).ti,ab,kf.
46.	(met analy* or metanaly* or technology assessment* or HTA or HTAs or technology overview* or technology appraisal*).ti,ab,kf.
47.	(meta regression* or metaregression*).ti,ab,kf.
48.	(meta-analy* or metaanaly* or systematic review* or biomedical technology assessment* or bio-medical technology assessment*).mp,hw.
49.	(medline or cochrane or pubmed or medlars or embase or cinahl).ti,ab,hw.
50.	(cochrane or (health adj2 technology assessment) or evidence report).jw.
51.	(comparative adj3 (efficacy or effectiveness)).ti,ab,kf.

52.	(outcomes research or relative effectiveness).ti,ab,kf.
53.	((indirect or indirect treatment or mixed-treatment or bayesian) adj3 comparison*).ti,ab,kf.
54.	(multi* adj3 treatment adj3 comparison*).ti,ab,kf.
55.	(mixed adj3 treatment adj3 (meta-analy* or metaanaly*)).ti,ab,kf.
56.	umbrella review*.ti,ab,kf.
57.	(multi* adj2 paramet* adj2 evidence adj2 synthesis).ti,ab,kf.
58.	(multiparamet* adj2 evidence adj2 synthesis).ti,ab,kf.
59.	(multi-paramet* adj2 evidence adj2 synthesis).ti,ab,kf.
60.	or/38-59
61.	28 and 37 and 60

EMBASE

Set	Search Statement
1.	exp Anxiety/
2.	(anxiety or anxieties or anxious or anxiousness).tw,kf.
3.	Anxiety Disorders/
4.	anxiety disorder?.tw,kf.
5.	anxiety state?.tw,kf.
6.	Panic Disorder/
7.	(panic adj (disorder? or attack?)).tw,kf.
8.	Agoraphobia/
9.	Agoraphobia?.tw,kf.
10.	exp Phobic Disorders/
11.	claustrophobia?.tw,kf.
12.	(phobia? or phobic).tw,kf.
13.	Phobia, Social/
14.	social anxiety disorder?.tw,kf.
15.	(fear* adj3 fall*).tw,kf.
16.	or/1-15
17.	aging/
18.	aged/
19.	"aged, 80 and over"/

20.	"frail elderly"/
21.	"health services for the aged"/
22.	"homes for the aged"/
23.	geriatrics/
24.	((old or retired) adj2 (people* or patient* or inpatient* or in-patient* or outpatient* or out-patient* or client* or person* or individual* or wom#n or man or men or age)).tw,kw.
25.	(aging or ageing or elder* or older* or geriatr* or gerontolog* or senior* or senescen* or retiree* or sexagenarian* or septuagenarian* or octagenarian* or nonagenarian* or centenarian* or supercentenarian* or veteran*).tw,kw,jw.
26.	(aged care or aged adult? or aged people or aged patient? or aged person? or aged women or aged men or aged individual?).tw,kf.
27.	or/17-26
28.	16 and 27
29.	Risk factor.mp. or exp Risk Factors/
30.	exp Risk Factors/
31.	((prevention or risk or protect* or mediat* or moderat*) adj2 (assess* or factor*)).tw,kw.
32.	exp Risk assessment/
33.	risk stratification.mp.
34.	risk modelling.tw.
35.	risk factors.tw.
36.	(predict* or influenc* or determinant* or associat* or correlat*).tw,kw.
37.	or/29-36
38.	(systematic review or meta-analysis).mp. [mp=title, abstract, heading word, drug trade name, original title, device manufacturer, drug manufacturer, device trade name, keyword heading word, floating subheading word, candidate term word]
39.	meta-analysis/ or systematic review/ or systematic reviews as topic/ or meta-analysis as topic/ or "meta analysis (topic)"/ or "systematic review (topic)"/ or exp technology assessment, biomedical/ or network meta-analysis/
40.	((systematic* adj3 (review* or overview*)) or (methodologic* adj3 (review* or overview*))).ti,ab,kf.
41.	((quantitative adj3 (review* or overview* or synthes*)) or (research adj3 (integrati* or overview*))).ti,ab,kf.

42.	((integrative adj3 (review* or overview*)) or (collaborative adj3 (review* or overview*)) or (pool* adj3 analy*)).ti,ab,kf.
43.	(data synthes* or data extraction* or data abstraction*).ti,ab,kf.
44.	(handsearch* or hand search*).ti,ab,kf.
45.	(mantel haenszel or peto or der simonian or dersimonian or fixed effect* or latin square*).ti,ab,kf.
46.	(met analy* or metanaly* or technology assessment* or HTA or HTAs or technology overview* or technology appraisal*).ti,ab,kf.
47.	(meta regression* or metaregression*).ti,ab,kf.
48.	(meta-analy* or metaanaly* or systematic review* or biomedical technology assessment* or bio-medical technology assessment*).mp,hw.
49.	(medline or cochrane or pubmed or medlars or embase or cinahl).ti,ab,hw.
50.	(cochrane or (health adj2 technology assessment) or evidence report).jw.
51.	(comparative adj3 (efficacy or effectiveness)).ti,ab,kf.
52.	(outcomes research or relative effectiveness).ti,ab,kf.
53.	((indirect or indirect treatment or mixed-treatment or bayesian) adj3 comparison*).ti,ab,kf.
54.	(multi* adj3 treatment adj3 comparison*).ti,ab,kf.
55.	(mixed adj3 treatment adj3 (meta-analy* or metaanaly*)).ti,ab,kf.
56.	umbrella review*.ti,ab,kf.
57.	(multi* adj2 paramet* adj2 evidence adj2 synthesis).ti,ab,kf.
58.	(multiparamet* adj2 evidence adj2 synthesis).ti,ab,kf.
59.	(multi-paramet* adj2 evidence adj2 synthesis).ti,ab,kf.
60.	or/38-59
61.	28 and 37 and 60

PsycINFO

Set	Search Statement
1.	exp Anxiety/
2.	(anxiety or anxieties or anxious or anxiousness).tw.
3.	Anxiety Disorders/
4.	anxiety disorder?.tw.

5.	anxiety state?.tw.
6.	Panic Disorder/
7.	(panic adj (disorder? or attack?)).tw.
8.	Agoraphobia/
9.	Agoraphobia?.tw.
10.	exp Phobias/
11.	claustrophobia?.tw.
12.	(phobia? or phobic).tw.
13.	Social Phobia/
14.	social anxiety disorder?.tw.
15.	(fear* adj3 fall*).tw.
16.	or/1-15
17.	aging/
18.	aged/
19.	Geriatric patients/
20.	Elder care/
21.	Older adulthood/
22.	pensioner.tw.
23.	geriatrics/
24.	((old or retired) adj2 (people* or patient* or inpatient* or in-patient* or outpatient* or out-patient* or client* or person* or individual* or wom#n or man or men or age)).tw.
25.	(aging or ageing or elder* or older* or geriatr* or gerontolog* or senior* or senescen* or retiree* or sexagenarian* or septuagenarian* or octagenarian* or nonagenarian* or centenarian* or supercentenarian* or veteran*).tw,jw.
26.	(aged care or aged adult? or aged people or aged patient? or aged person? or aged women or aged men or aged individual?).tw.
27.	or/17-26
28.	16 and 27
29.	Risk factor.mp. or exp Risk Factors/
30.	exp Risk Factors/
31.	((prevention or risk or protect* or mediat* or moderat*) adj2 (assess* or factor*)).tw.
32.	exp Risk assessment/
33.	risk stratification.mp.

34.	risk modelling.tw.
35.	risk factors.tw.
36.	(predict* or influenc* or determinant* or associat* or correlat*).tw.
37.	or/29-36
38.	(systematic review or meta-analysis).mp. [mp=title, abstract, heading word, table of contents, key concepts, original title, tests & measures, mesh word]
39.	meta-analysis/ or systematic review/ or systematic reviews as topic/ or meta-analysis as topic/ or "meta analysis (topic)"/ or "systematic review (topic)"/ or exp technology assessment, biomedical/ or network meta-analysis/
40.	((systematic* adj3 (review* or overview*)) or (methodologic* adj3 (review* or overview*))).ti,ab.
41.	((quantitative adj3 (review* or overview* or synthes*)) or (research adj3 (integrati* or overview*))).ti,ab.
42.	((integrative adj3 (review* or overview*)) or (collaborative adj3 (review* or overview*)) or (pool* adj3 analy*)).ti,ab.
43.	(data synthes* or data extraction* or data abstraction*).ti,ab.
44.	(handsearch* or hand search*).ti,ab.
45.	(mantel haenszel or peto or der simonian or dersimonian or fixed effect* or latin square*).ti,ab.
46.	(met analy* or metanaly* or technology assessment* or HTA or HTAs or technology overview* or technology appraisal*).ti,ab.
47.	(meta regression* or metaregression*).ti,ab.
48.	(meta-analy* or metaanaly* or systematic review* or biomedical technology assessment* or bio-medical technology assessment*).mp,hw.
49.	(medline or cochrane or pubmed or medlars or embase or cinahl).ti,ab,hw.
50.	(cochrane or (health adj2 technology assessment) or evidence report).mp. [mp=title, abstract, heading word, table of contents, key concepts, original title, tests & measures, mesh word]
51.	(comparative adj3 (efficacy or effectiveness)).ti,ab.
52.	(outcomes research or relative effectiveness).ti,ab.
53.	((indirect or indirect treatment or mixed-treatment or bayesian) adj3 comparison*).ti,ab.
54.	(multi* adj3 treatment adj3 comparison*).ti,ab.
55.	(mixed adj3 treatment adj3 (meta-analy* or metaanaly*)).ti,ab.
56.	umbrella review*.ti,ab.

57.	(multi* adj2 paramet* adj2 evidence adj2 synthesis).ti,ab.
58.	(multiparamet* adj2 evidence adj2 synthesis).ti,ab.
59.	(multi-paramet* adj2 evidence adj2 synthesis).ti,ab.
60.	or/38-59
61.	28 and 37 and 60
